# The protein fraction from wheat-based dried distiller's grain with solubles (DDGS): extraction and valorization

**DOI:** 10.1016/j.nbt.2015.01.007

**Published:** 2015-12-25

**Authors:** M.F. Villegas-Torres, J.M. Ward, G.J. Lye

**Affiliations:** The Advanced Centre for Biochemical Engineering, Department of Biochemical Engineering, University College London, Gordon Street, WC1H 0AH London, UK

## Abstract

Nowadays there is worldwide interest in developing a sustainable economy where biobased chemicals are the lead actors. Various potential feedstocks are available including glycerol, rapeseed meal and municipal solid waste (MSW). For biorefinery applications the byproduct streams from distilleries and bioethanol plants, such as wheat-based dried distiller's grain with solubles (DDGS), are particularly attractive, as they do not compete for land use. Wheat DDGS is rich in polymeric sugars, proteins and oils, making it ideal as a current animal feed, but also a future substrate for the synthesis of fine and commodity chemicals. This review focuses on the extraction and valorization of the protein fraction of wheat DDGS as this has received comparatively little attention to date. Since wheat DDGS production is expected to increase greatly in the near future, as a consequence of expansion of the bioethanol industry in the UK, strategies to valorize the component fractions of DDGS are urgently needed.

## Highlights

• Wheat DDGS is a major by-product from first generation bioethanol facilities.
• Variability in feedstocks and processing generates inconsistent batches of wheat DDGS impeding protein prediction.
• We described possible methods for extraction of gluten from wheat DDGS.
• We discuss options for valorization of the protein fraction from wheat DDGS into chemicals or biomaterials.

Agenda 21 was established in 1992 and described an action plan towards the consolidation of a green economy [1]. One of the program goals was to increase the availability of renewable raw materials and the development of biotechnological processes for the production of sustainable biomass-based chemicals. These could replace petroleum-based resources and/or expand the range of accessible compounds at industrial scale [2]. To achieve this, it is necessary to: (1) understand the various biomass feedstocks available for such purposes; (2) improve and develop biorefinery-basic technologies for the fractionation of raw material; and (3) improve and develop conversion methods of each fraction towards chemical production [2,3].

In 2007 the UK Government established the Industrial Biotechnology Innovation and Growth Team (IB-IGT) to identify opportunities (and challenges) for future competitiveness in industrial Biotechnology (IB). In 2009, their report ‘*IB 2025: Maximising UK Opportunities from Industrial Biotechnology in a Low Carbon Economy*’ identified five crucial recommendations towards the consolidation of IB in the UK. These included: to speedup IB knowledge and innovation transfer; to attract and retain IB experts in science, engineering and management; and to create a supportive environment at both public and private level. The team also estimated that up to £12 billion per year could be added to the UK economy from IB innovation [4,5].

To date, several bio-based chemicals have been successfully produced at industrial scale from various raw materials, for example, biodiesel from plant oil and bioethanol from sucrose and starch. However, the use of byproduct streams is of special interest as they do not interfere with the debate on alternative land uses. Their composition can be highly variable depending on the source, but generally they are composed of oils, polysaccharides, lignin and/or proteins. Due to the high content of polysaccharides and lignin across different sources most of the recent research has focused on valorization of these fractions [6]. In contrast, little attention has been paid to the protein fraction, except for its nutritional value as animal feed. Various byproduct streams that contain high quantities of protein, that is, >10% (w/w), come from the following industries: (1) distilleries and first generation biofuel production, that is, DDGS; (2) agriculture, such as stover, straw, leaves and hay; and (3) oil and biodiesel production, that is, meals and seedcakes. Their protein content can vary from as little as 3% (w/w) found in maize stover, to 65% (w/w) present in jatropha seed meal [7].

Among the above feedstocks, wheat DDGS is of particular importance to the UK after the recent establishment of two wheat-bioethanol refineries; one by Ensus, established in 2010 with a maximum capacity of 400,000 m^3^ of bioethanol and 350,000 ton of DDGS per year, and one by Vivergo, established in 2013 with a maximum production of 420,000 m^3^ of bioethanol and 500,000 ton per year. Before 2010, there was an annual production of 250 kt/annum of wheat DDGS from the UK distillery industry. This will increase at least fourfold by the time these two bioethanol plants are fully operational, and is expected to rise even further if more companies build plants in the UK, such as Vireol: planned for 2016 with a capacity of 200 million liters per year [8,9]. This increased supply is expected to saturate the animal feed market resulting in a lower value for this feedstock. The UK Government thus has an interest to identify alternative uses and opportunities for valorization of wheat DDGS.

**Figure 1:**
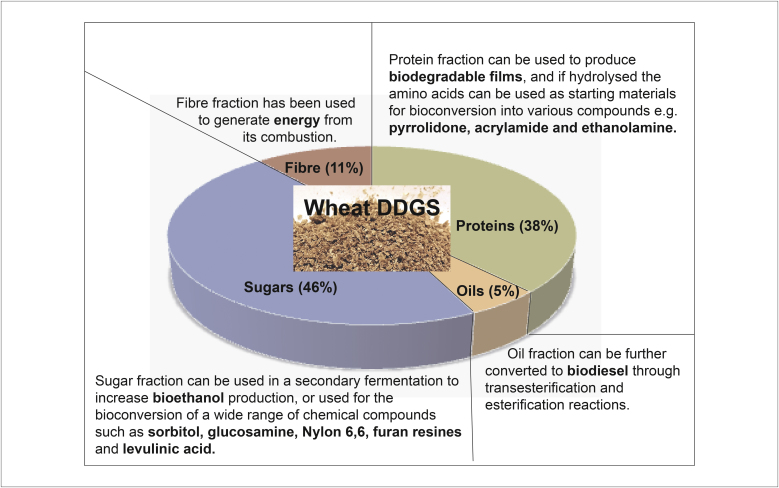
Wheat DDGS composition and options for valorization of the different fractions.

Wheat DDGS is composed of polymeric sugars (cellulose and hemicellulose), oils, protein and other materials for example, ash. Their proportion varies according to the production process and the wheat variety initially used, but on average is 46%, 5%, 38% and 11% (w/w), respectively [10]. Each fraction can be further valorized (Fig. 1), yet despite the wide range of compounds that can be obtained from it, the protein fraction has received limited attention to date. This review therefore focuses on protein extraction and valorization opportunities from wheat-based DDGS as a UK priority.

## Wheat proteins and varieties used in UK distilleries

Wheat protein is mainly composed of gluten (80–85% (w/w) of total protein), while the remaining fraction comprises a heterogeneous group of globulins and albumins. The latter are soluble in aqueous salt solutions, and in their monomeric forms are <25 kDa except for a very small portion that can reach up to 70 kDa. In contrast, gluten proteins are soluble in ethanol due to the high content of non-polar and/or uncharged side chain amino acids. When solubilized, two main fractions are recognized: (1) monomeric gliadins of around 30–80 kDa; and (2) glutenin subunits (GS) that can vary between 80 and several million kDa [11].

GS are more difficult to solubilize than gliadins as the various subunits are bound through disulfide-bonds. For their solubilization, a reducing pre-treatment is thus required. In solution, low molecular weight (LMW-GS) and high molecular weight (HMW-GS) subunits can be differentiated [11]. The weight ratio HMW-GS to LMW-GS varies between 0.18 and 0.74, indicating that LMW-GS are in a molar excess to HMW-GS, possibly due to the size difference and the number of coding genes within the genome [12].

**Figure 2:**
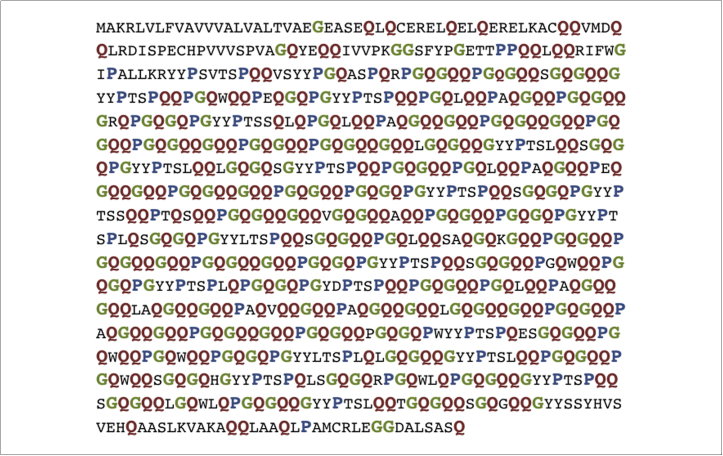
The amino acid sequence of a glutenin from wheat showing a biased amino acid composition towards glutamine (35% of the total amino acid sequence highlighted in red), glycine (20% highlighted in green) and proline (13% highlighted in blue) The illustrated protein is from *Triticum aestivum* with the GenBank accession number: CAA27052.1. This example is 838 amino acids in length.

There are more than 20 different HMW-GS varying among wheat varieties. Within each variety, there are normally three to five different HMW-GS expressed simultaneously. They are all biased in their amino acid composition towards glutamine, proline and glycine, which are present in repetitive sequences within the central domain of the protein (Fig. 2) forming overlapping β-reverse-turns. At the ends of this sequence, cysteine residues are present to form disulfide bonds introducing some flexibility to the central rigid structure. An example of a wheat glutenin is shown in Fig. 2. This particular protein has 292 glutamine residues (35% of the total amino acids in the protein), 164 glycine (20%), 107 proline (13%) and 46 tyrosine (5%) residues. In the case of LMW-GS, 40 different variants have been identified and in one single variant 7-16 different LMW-GS can be expressed simultaneously. LMW-GS is rich in glutamine and proline located in repetitive sequences at the N-terminal, forming β-reverse turns; and the non-repetitive sequences at the C-terminal forms an α-helical secondary structure. Within this domain at least six cysteine residues are found, which are responsible for the globular structure of the C-terminal in comparison to the N-terminal [11].

In the UK different soft milling wheat varieties are used for distilling: Beluga, Viscount, Zebedee, Invicta, Glasgow, Cassius, Rodigus, Istabraq, Alchemy, Claire, Scout, and Warrior [13]. Their main characteristics are their softness for milling and their low protein content because of the inverse relationship between protein and starch content in wheat seed [14]. On average the total protein percentage in each variety is between 10 and 12% (w/w) (Source: HGCA – Cereals and Oilseeds Division of the Agriculture and Horticulture Development Board (AHDB)), but the percentage of a particular type/group of proteins in each variety has not been reported to date. In addition, wheat varieties are normally mixed for bioethanol production [15], thus it is not possible to reliably predict the distribution of different proteins in the resultant wheat DDGS.

## Wheat DDGS protein and its extraction

The wheat DDGS protein fraction is not only composed of wheat protein, namely gluten, globulins and albumins, but also of yeast protein. This is because following the fermentation step the recovered DDGS is normally mixed, and dried, with the light concentrated slurry to improve its animal feed value. The contribution of yeast protein to wheat DDGS is not well documented, but previous studies have shown that wheat fermentation for the production of bioethanol or beer does not affect the amino acid composition of DDGS [16]. In the case of corn-based DDGS, however, up to 20% (w/w) yeast protein has been reported in the total protein content [17]. In the same study, an exhaustive analysis of the amino acid distribution was performed throughout corn processing including yeast, ground corn and corn DDGS. The results indicated that some amino acids increased, others decreased and others remained unchanged, but that their individual contribution is not higher than 2%. It thus appears that despite the contribution of yeast protein, the overall amino acid distribution is unaltered [17].

Wheat processing for beer/bioethanol and DDGS production will influence protein degradability. This is due to enzymatic modification during fermentation and/or the DDGS heat-drying step during beer/bioethanol processing [18,19]. Since there is no change in amino acid composition it can be expected that the solubility characteristics of the wheat proteins are unaltered. Consequently, alcohol-based solvents could be implemented to solubilize the gluten, as well as a reduction step before extraction to disrupt the disulfide bonds.

While there have been numerous reports on the processing of milled seed for the (selective) extraction of protein, to the best of our knowledge there have been none on the use of wheat DDGS for such purposes [20–22]. In the case of the milled wheat seed there is generally an initial step for albumin and globulin extraction carried out using a 0.5 m NaCl solution [22]. If albumin and globulin are to be separated, then a pre-treatment step using deionized water must be implemented to first extract the albumin [20]. The gliadin fraction is then obtained using 70% (v/v) aqueous ethanol, while the glutenin is extracted using 50% (v/v) 1-propanol plus 1% (v/v) dithiothreitol (DTT) [20,22]. In a separate study a single step extraction using 50% (v/v) 1-propanol mixed with 1% (v/v) DTT at 60°C was used to obtain a mixture of gliadins and glutenins of different molecular weights [21].

A type of DDGS that has received some attention for protein extraction, particularly in the United States [23], is maize-based DDGS. In contrast to wheat, maize protein is composed of: zein, glutelin, globulin and albumin. Among these four protein classes, zein is the equivalent to wheat gliadin however it is deficient in essential amino acids, with a bias towards glutamic acid, leucine, proline and alanine. It is soluble in aqueous alcohol, urea, high pH or anionic detergents. Zein, like gliadin, arranges in globular structures linked together by disulfide bonds between different zein peptides of various length, solubility and charge.

Zein has been extracted direct from maize since 1939, mainly employing aqueous ethanol solutions between 50 and 90% (v/v). Besides this method, pH variation and hydrolytic enzymes have also been implemented in protein extraction from maize kernel. pH values lower than 1, using HCl, or higher than 12, using NaOH, are normally applied, but such pH variations can degrade the protein. Proteases have proven to be an alternative method [24], but enzyme costs limit the industrial application of this approach. Analogous methods have been described when using maize DDGS as feedstock for zein extraction. Some used NaOH addition and elevated temperatures [25,26], while others employed ethanol-based extraction with or without the addition of NaOH after a reductive pre-treatment step. In addition, direct enzyme extraction using a mixture of proteases has also been performed [23]. In each case more than 70% (w/w) of the protein has been solubilized.

Considering the above comparison, it is clear that gluten extraction from wheat, and zein extraction from maize kernel, or maize DDGS, are performed using comparable methodologies. It is therefore reasonable to expect that protein fractionation from wheat DDGS can be performed using equivalent methods to those already published using maize and similar yields can be expected. Thus, an average of 265 kton/year of extracted protein would be initially available to produce high-value products (based on a 1000 kton/year of DDGS production from Ensus and Vivergo, and a 70% protein extraction efficiency).

For protein extraction at large scale, as opposed to analytical and laboratory scales, it will also be necessary to consider the environmental impact of the process and its sustainability. This will restrict the choice of chemicals used and the energy requirements of individual unit operations. The predicted increases in wheat DDGS production in the UK suggest it is now imperative to optimize these methods and also to consider options for valorization of the protein fraction in this important biorefinery feedstock.

## Wheat DDGS protein and hydrolysate valorization

Up to now, only one initial attempt to produce glutamic acid from wheat protein, as a representation of wheat DDGS valorization, has been reported in the literature [27]. Glutamic acid is considered one of the top twelve building block [28] chemicals as it can be converted into diols, diacids and aminodiols, which are monomers of polyesters and polyamides widely employed as polymers [28]. The current production process of glutamic acid requires a neutralization step with further purification and conversion from the sodium salt to the free amino acid with high environmental and financial costs [28]; thus, alternative production processes are required. In the above-mentioned report, Sari et al. [27] tested acid hydrolysis, enzymatic hydrolysis and their combination to obtain glutamic acid. According to their results, the most sustainable method included enzymatic hydrolysis, using a mixture of Validase FP Concentrate and Peptidase R, followed by a diluted acid hydrolysis giving an 80% (w/w) yield. Consequently, this work supports the idea of using wheat DDGS protein as feedstock for the production of commodity and fine chemicals of industrial relevance.

Besides glutamic acid, various amino acids have proven to be useful in biocatalytic syntheses. For example: l-phenylalanine has been converted to cinnamic acid, by a deamination step, and can be further decarboxylated to synthesize styrene; likewise l-lysine has been employed in the conversion of 1,5-diaminopentane, caprolactam, and 5-amino valeric acid [29]. These studies suggest that other amino acids, and not just glutamic acid and glutamine, are of interest when designing wheat DDGS protein valorization strategies.

Wheat DDGS protein presents a biased amino acid composition (Fig. 2) where glutamine (residues in highlighted in red) is in the highest proportion, followed by proline (residues in highlighted in blue), leucine, aspartic acid, phenylalanine, valine, serine, arginine, and glycine (residues in highlighted in green) [10] as previously mentioned. Among them, leucine, phenylalanine, and valine are essential amino acids while the others are considered non-essential. To maintain the feed value of wheat DDGS, the non-essential amino acids can be further valorized, while the essential ones should remain in the residual material [6,29].

Proline can be chemically transformed to pyrrolidone, a precursor of *N*-vinylpyrrolidone, a 15,000 €/ton polymer used in the cosmetic and medical sector. Aspartic acid can give rise to acrylamide used in wastewater treatment [30], and to maleic and fumaric acid employed in the food and paper industries in salt form as chelating agents and sweeteners [28,30]. Serine, after an α-decarboxylation step, produces ethanolamine used in several industries with an annual demand of several hundred kilotonnes. l-Arginine has been hydrolyzed and decarboxylated to 1,4-diaminobutane, a 4,6-nylon precursor [29] while glycine can be converted to oxalic acid used as bleaching agent in the textile and pulp industries and in wastewater treatment [30]. Hence, there would appear to be significant potential for the valorization of non-essential amino acids present in wheat DDGS.

One of the main bioprocess limitations to achieving valorization of all the non-essential amino acids is their separation after hydrolysis. They can be separated by size, charge and hydrophobic characteristics; differential reactivity with other chemicals producing compounds that are easier to separate [29]; or a process of electrodialysis coupled to chemical or enzymatic conversion which is currently under development. Nevertheless, further work is required, as the above methods would not be cost-effective at industrial scale at present [7].

An alternative strategy to valorize wheat DDGS protein without prior hydrolysis and purification steps is to employ the protein and/or extracted peptides in the manufacture of protein-based biomaterials. Different proteins have been employed in the manufacture of biopolymers such as wheat, zein, casein and soy. They are biodegradable, can increase soil fertility, and are easily disposable. As a result, they are important as packing materials, and can also be employed in coating industries and agriculture. To date they have been successfully employed in the synthesis of edible food packing, but further work is required to improve film properties aimed at mimicking those of synthetic ones. This has proven to be a major challenge due to limitations over the control of bond formation and the quality of protein crosslinking [31].

Wheat biopolymers are characterized by their elastic behavior, and are efficient barriers for oxygen, carbon dioxide and aromatic compounds; however, their mechanical properties are not ideal. Thermoformation [32], plasticizers [33], and UV crosslinking [31] have been successfully applied in their manufacture. Material elasticity is inversely correlated to the level of crosslinking translated into the number of S—S bonds present as demonstrated by Pallos et al. [32]. In this study gluten was mixed with non-cereal proteins that contained higher amounts of cysteine residues, resulting in a material with reduced elasticity and increased disulfide bonds. Mixtures including acids, saturated fatty acids, or formaldehyde affect the biomaterial properties in terms of elasticity and water vapor permeability [32]. An interesting biopolymer blend is the wheat gluten/methylcellulose film. This particular mixture has shown improved properties at different ranges depending on the proportion of each component: higher moisture barrier, tensile strength and better water vapor permeability [33].

To date, the use of wheat DDGS extracted protein in polymerization processes has not been reported. Nevertheless, its potential use is clear, as it would be a cheap gluten source. During protein extraction, the solubilization of other materials such as lignin, cellulose, and hemicellulose would affect the biomaterial properties, as it was previously reported in the case of methylcellulose [33]. Additionally, the DDGS production process and the extraction methods used can be expected to have an impact on protein structure [11] and its subsequent polymerization. Hence it may be worth investigating film formation from wheat DDGS extracted protein, and how the extraction conditions affect the mechanical properties of the resulting biopolymer.

## Conclusions

Previous studies employing wheat DDGS protein have mainly focused on analyzing its nutritional value as animal feed. However, there is now growing interest in valorization of the protein fraction into a range of potential products. To progress this research, and to establish industrial processes for the manufacture of protein-based chemicals and biomaterials, efficient protein extraction methods must be established. This review suggests that rapid initial progress could be made by considering previous research on protein extraction from maize DDGS. Once the protein is extracted there appear to be numerous opportunities for its valorization including the extraction of individual amino acids with the potential to be transformed into building blocks for polymers or, pharmaceuticals, or the solubilization of the whole or partially hydrolyzed protein for use in the formation of wheat-based films. In either case it will require the development of new chemical and biological processing techniques that are the focus of our current research.
